# The *IGF1* P2 promoter is an epigenetic QTL for circulating IGF1 and human growth

**DOI:** 10.1186/s13148-015-0062-8

**Published:** 2015-03-13

**Authors:** Meriem Ouni, Yasemin Gunes, Marie-Pierre Belot, Anne-Laure Castell, Delphine Fradin, Pierre Bougnères

**Affiliations:** Institut National de la Santé et de la Recherche Médicale U986, Bicêtre Hospital, Paris Sud University, 80 rue du Général Leclerc Le Kremlin-Bicêtre, Paris, 94276 France; Department of Pediatric Endocrinology and Diabetes, I3E Pole, Bicêtre Hospital, Paris Sud University, rue du Général Leclerc Le Kremlin-Bicêtre, Paris, 94276 France

**Keywords:** QTL^epi^, Growth, Short stature, Height, IGF1, Epigenetics, DNA methylation, t-DMR

## Abstract

**Background:**

Even if genetics play an important role, individual variation in stature remains unexplained at the molecular level. Indeed, genome-wide association study (GWAS) have revealed hundreds of variants that contribute to the variability of height but could explain only a limited part of it, and no single variant accounts for more than 0.3% of height variance. At the interface of genetics and environment, epigenetics contributes to phenotypic diversity. Quantifying the impact of epigenetic variation on quantitative traits, an emerging challenge in humans, has not been attempted for height. Since insulin-like growth factor 1 (IGF1*)* controls postnatal growth, we tested whether the CG methylation of the two promoters (P1 and P2) of the *IGF1* gene is a potential epigenetic contributor to the individual variation in circulating IGF1 and stature in growing children.

**Results:**

Child height was closely correlated with serum IGF1. The methylation of a cluster of six CGs located within the proximal part of the *IGF1* P2 promoter showed a strong negative association with serum IGF1 and growth. The highest association was for CG-137 methylation, which contributed 13% to the variance of height and 10% to serum IGF1. CG methylation (studied in children undergoing surgery) was approximately 50% lower in liver and growth plates, indicating that the *IGF1* promoters are tissue-differentially methylated regions (t-DMR). CG methylation was inversely correlated with the transcriptional activity of the P2 promoter in mononuclear blood cells and in transfection experiments, suggesting that the observed association of methylation with the studied traits reflects true biological causality.

**Conclusions:**

Our observations introduce epigenetics among the individual determinants of child growth and serum IGF1. The P2 promoter of the *IGF1* gene is the first epigenetic quantitative trait locus (QTL^epi^) reported in humans. The CG methylation of the P2 promoter takes place among the multifactorial factors explaining the variation in human stature.

**Electronic supplementary material:**

The online version of this article (doi:10.1186/s13148-015-0062-8) contains supplementary material, which is available to authorized users.

## Background

Although defined as a variant of normal, ‘idiopathic’ short stature in a child is a common source of medical investigation and a potential indication for treatment with growth hormone [1]. The molecular causes of idiopathic short stature are multiple [2], as expected for a multifactorial trait that is influenced both by individual genotypes and environmental factors [3].

First studied by F. Galton at the end of the nineteenth century [4], height heritability and variability remain partially understood [5,6]. Twin and family studies have consistently estimated that the additive genetic contribution to normal variation in adult height approximates 80% in a given population at a given time [7-9]. As predicted by R.A Fisher [7], the genetic heritability of height is explained by many variants of individually small effect. A recent genome-wide association study (GWAS) study identified variants at 180 loci that together explain approximately 12% of the heritable variation in height [10]. Another GWAS suggested that half of the heritability of height can be accounted for by additive effects of a large number of common variants [11]. The most recent meta-analysis using the summary statistics from 79 studies totalling 253,288 individuals of European ancestry showed that the most strongly associated 2,000, 3,700, and 9,500 SNPs account for 21%, 24%, and 29% of height variance, respectively [9]. No single variant identified in these GWAS explained more than 0.3% of height variance. Genetics thus seems to explain a major but limited part of height variability across individuals.

Classically, quantitative geneticists envision DNA sequence variants as the only source of heritable phenotypes. This view should be revised in light of accumulating evidence for widespread epigenetic variation in natural populations [12,13]. Indeed, the current sequence-based quantitative trait locus (QTL) approaches for dissecting complex traits miss important phenotypic effects exerted by epigenetic variants [13]. While epigenetics is a considerable source of inherited and acquired variability among humans [14], its contribution to height variability has not been studied.

To explore the individual variability of child growth and search for new causes of short stature, we selected the *insulin-like growth factor 1* (*IGF1*) gene as a prominent physiological candidate. IGF1 is a key player in postnatal growth and GH signaling. Inactivating mutations in the *IGF1* gene alter postnatal growth in humans [15] and mice [16]. Clinical studies show a strong correlation between height and circulating IGF1 in childhood [17], and IGF1 production disorders are a source of short stature [18]. However, common genetic variation in the *IGF1* gene sequence does not contribute significantly to adult height variation in populations of Caucasians [19,20] while it does so in Asians whose *IGF1* allele frequency is different [21,22].

Twin studies indicate that the genetically determined proportion of circulating IGF1 variance ranges between 38% and 80% [23-25]. The association of circulating IGF1 with several genetic variants is debated [26-28], but variants at the *IGF1* locus do not seem to influence circulating IGF1 in Caucasian adults [26,29] except, maybe, the commonest Z allele of the microsatellite located 1 kb upstream of the *IGF1* gene [19,30]. Overall, the genetic basis for serum IGF1 variability remains unknown in adults and has not been studied in growing children.

Most epigenetic effects on phenotypes result from effects on gene expression, particularly for the methylation of CG located within gene promoters. Our hypothesis was that variation in epigenetic marks located in the promoters that regulate *IGF1* gene expression [31] might play a role in modulating *IGF1* gene expression, thus contribute to the individual variation of IGF1 production and child growth. In addition, these promoters are CG-poor and expected to exhibit inter-individual variation [32]. Instead of using a whole-methylome array to test associations with height or serum IGF1, our candidate gene approach enabled us to quantify the methylation of each CG of the *IGF1* promoters accurately. Indeed, an individual CG may have a significant functional role different from its CG neighbors [33,34], and this effect cannot be unraveled if the specific CG is not part of the commercial array. In addition, quantifying the methylation of each CG provides a mean to test its correlation with height or circulating IGF1.

Among the various categories of *IGF1* transcripts, class I transcripts have their initiation sites on exon 1 and are driven by P1 promoter, while class II transcripts use exon 2 as a leader exon and are driven by P2 promoter [35,36]. *IGF1* transcripts initiating at P1 are constitutively expressed in many tissues, transcripts initiating at P2 are expressed primarily but not exclusively in the liver [31,37].

The current study explores the relation between the methylation of the CGs located within *IGF1* gene promoters and child height and circulating IGF1 and tests how promoter methylation affects transcriptional activity in subjects’ blood cells and in transfected cells.

## Results

### Patterns of methylation in the two IGF1 gene promoters

**Figure 1:**
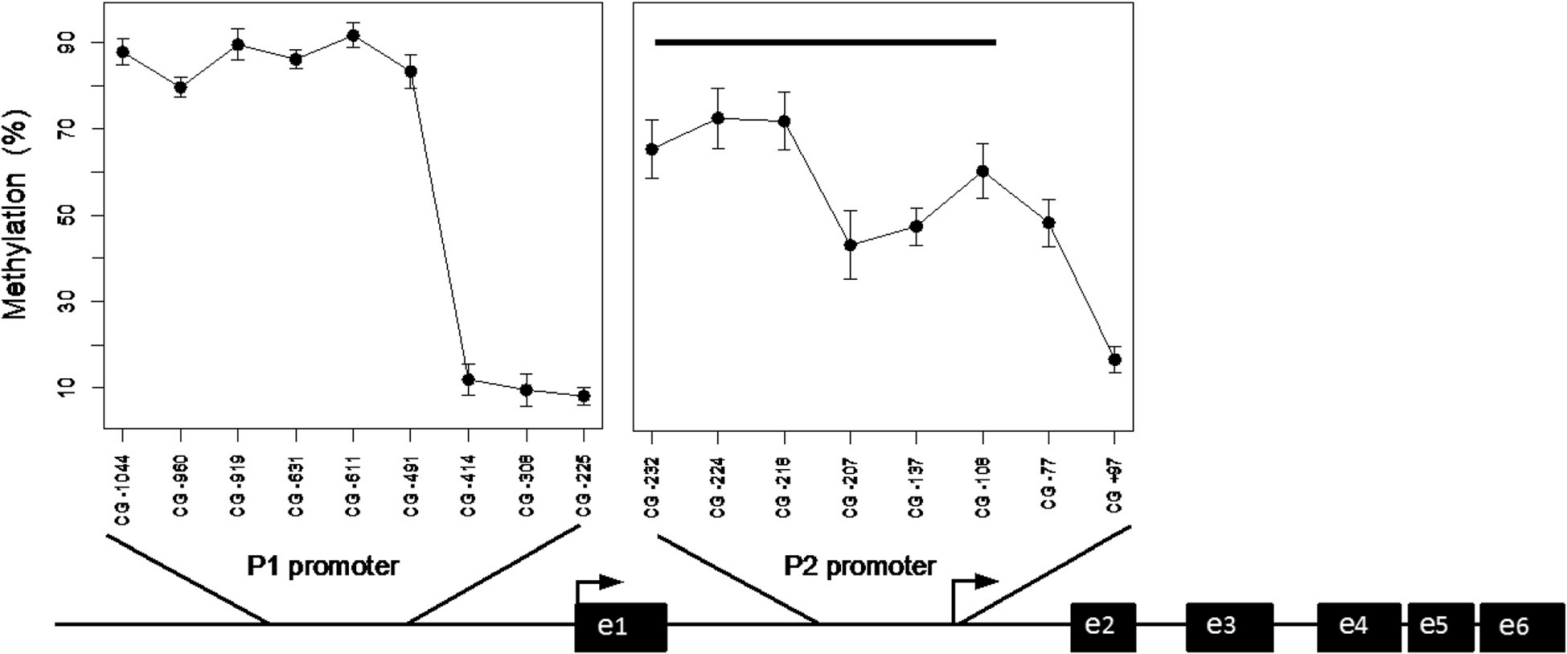
**The two** ***IGF1*** **gene promoters (P1 and P2) are figured in the lower part of the figure (broken arrows show the TSS of each promoter).** The upper part of the figure shows the methylation level of each studied CG in the 216 children (mean ± SD). The horizontal black bar encompasses the cluster of six CGs of P2 promoter whose methylation is inversely correlated with height and serum IGF1.

The patterns of CG methylation within the two promoters of *IGF1* are depicted in Figure 1. Methylation in the proximal part of P2 promoter showed an important individual variability (Figure 1 and Additional file 1: Table S2). Strong intra-promoter correlations (10^−8^ < *P* < 10^−3^) were observed between methylation of the CGs in P2 promoter, while there was no correlation within P1 or between CGs of P1 and P2. The methylation at the P2 and P1 promoters does not seem to be significantly influenced by sex or age (Additional file 1: Table S3).

**Figure 2:**
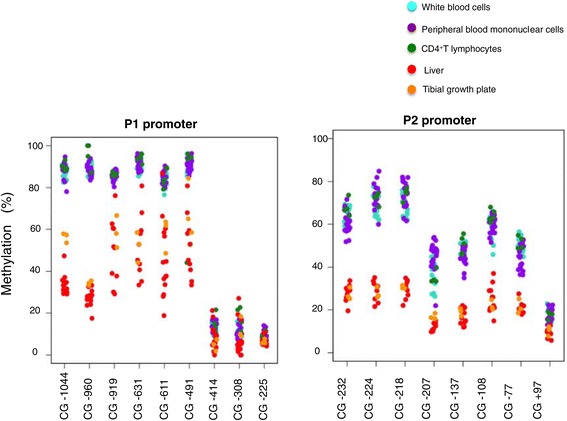
**Tissue-specific methylation levels in white blood cells (WBC), peripheral blood mononuclear cells (PBMC), CD4** ^**+**^ **T lymphocytes, liver, and tibial growth plates.** For simplicity, values in only ten patients are figured for WBC and PBMC. The complete set of values is shown in Additional file 1: Table S4.

Methylation levels were comparable in white blood cells (WBC), peripheral blood mononuclear cells (PBMC), and CD4^+^ T lymphocytes (Figure 2). The methylation levels were approximately 50% lower for most P1 and P2 CGs in liver and growth plates than in blood cells (Figure 2, Additional file 1: Table S4). The *IGF1* promoters can thus be considered as tissue differentially methylated regions (t-DMR).

### Height and serum IGF1 are inversely correlated with CG methylation of the P2 promoter

**Figure 3:**
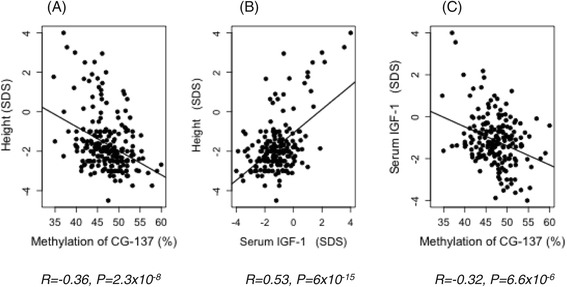
**CG-137 methylation correlates negatively with height and with serum IGF1. (A)** Inverse correlation between CG-137 methylation and height in studied children (*N* = 216), (*Y* = −0.12X + 4.1; *R* = −0.36, *P* = 2.3 × 10^−8^). **(B)** Correlation between circulating IGF1 and height (*N* = 184), *Y* = 0.6X − 1 (*R* = 0.53, *P* = 6 × 10^−15^). **(C)** Inverse correlation between CG-137 methylation and serum IGF1 (*N* = 184), (*Y* = −0.09X + 3.2; *R* = −0.32, *P* = 6.6 × 10^−6^).

In the discovery cohort of 110 children (Table 1), height was found to be inversely correlated with the methylation of 4/8 CGs of the P2 promoter (10^−3^ < *P* < 0.01 after Bonferroni correction) (Table 1). These observations were replicated in a second cohort of 106 children (Table 1). After merging the two cohorts, the inverse correlation with height was confirmed for 6/8 CGs of the P2 promoter and was maximal for CG-137 methylation (*P* = 4 × 10^−7^) (Figure 3A and Table 1), so that CG-137 methylation accounted for 13% of height variability (Figure 3A). Since 6/8 CGs in P2 trend to an association and are physically located very close together, the average methylation was calculated for these 6 CGs and showed a strong inverse correlation with height (*P* = 7 × 10^−5^) (Table 1) and with only 1/9 CGs (CG-611) of the P1 promoter (*P* = 4.2 × 10^−7^) (Table 1).

**Table 1:** **Correlation between the percent methylation of each studied CG and child height (** ***N = 216*** **)**

		**Height (SDS)**					
		**Discovery cohort**		**Replication cohort**		**Total**	
		***R***	***P***	***R***	***P***	***R***	***P***
P1 promoter	CG-1044	0.02	1	0.02	1	0.03	1
	CG-960	0.05	1	0.06	1	0.06	1
	CG-919	0.02	1	−0.13	1	0.04	1
	CG-631	−0.04	1	−0.04	1	−0.09	1
	*CG-611*	*−0.25*	*0.1*	*−0.48*	*2.2.10* ^*−5*^	*−0.39*	*4.10* ^*−7*^
	CG-491	−0.06	1	−0.31	0.03	−0.10	1
	CG-414	0.00	1	−0.19	0.4	0.09	1
	CG-308	0.02	1	0.02	1	0.00	1
	CG-225	0.08	1	0.012	1	−0.10	1
P2 promoter	*CG-232*	−0.08	1	*−0.28*	*0.03*	*−0.21*	*0.01*
	*CG-224*	*−0.32*	*10* ^*−2*^	*−0.17*	*0.7*	*−0.24*	*0.003*
	*CG-218*	*−0.36*	*10* ^*−3*^	*−0.30*	*0.02*	*−0.33*	*1.2.10* ^*−5*^
	*CG-207*	*−0.35*	*2.10* ^*−3*^	*−0.12*	*1*	*−0.24*	*4.10* ^*−3*^
	*CG-137*	*−0.30*	*10* ^*−2*^	*−0.40*	*1.9.10* ^*−4*^	*−0.36*	*4.10* ^*−7*^
	CG-108	−0.25	0.10	−0.19	0.5	−0.23	0.12
	Average*	*−0.31*	*0.007*	*−0.27*	*0.004*	*−0.3*	*7.10* ^*−5*^
	CG-77	−0.09	1	−0.04	1	−0.04	1
	CG + 97	−0.08	1	−0.03	1	0.07	1

*Values for the averaged 6 CGs from −108 to −232. To account for multiple CG testing, we used Bonferroni correction of the *P* values. *P* values greater than 1 are equated to 1.

Italicized letters and numbers indicate significant correlation.

Height was closely correlated with serum IGF1 (*P* = 6 × 10^−15^) (Figure 3B), so that the variability of circulating IGF1 accounts for 27% of height variability in the current cohort. As previously observed for height, strong inverse correlations were found between serum IGF1 and the methylation of 4/8 CGs in the P2 promoter (10^−4^ < *P* < 0.01 after Bonferroni correction), notably CG-137 (Figure 3C and Table 2). CG-137 methylation contributed 10% to serum IGF1 variation. Again, CG-611 was the only CG of the P1 promoter to show an inverse correlation with serum IGF1 (*P* = 0.009) (Table 2).

**Table 2:** **Correlation between methylation of all studied CGs and serum IGF1 concentration (SDS) (** ***N = 184*** **)**

		**Serum IGF1 (SDS)**					
		**Discovery cohort**		**Replication cohort**		**Total**	
		***R***	***P***	***R***	***P***	***R***	***P***
P1 promoter	CG-1044	−0.05	1	0.08	1	0.03	1
	CG-960	0.08	1	0.07	1	0.01	1
	CG-919	0.07	1	−0.22	0.6	−0.10	1
	CG-631	−0.18	0.7	−0.07	1	−0.10	1
	*CG-611*	*−0.27*	*0.06*	*−0.33*	*0.02*	*−0.25*	*9.10* ^*-3*^
	CG-491	−0.05	1	−0.11	1	−0.07	1
	CG-414	0.06	1	0.21	0.6	−0.15	0.5
	CG-308	0.00	1	0.04	1	0.03	1
	CG-225	0.09	1	0.09	1	0.04	1
P2 promoter	*CG-232*	*−0.19*	*0.46*	*−0.29*	*0.08*	*−0.24*	*8.10* ^*−3*^
	*CG-224*	*−0.24*	*0.14*	*−0.05*	*1*	*−0.19*	*9.10* ^*−2*^
	*CG-218*	*−0.23*	*0.17*	*−0.22*	*0.5*	*−0.24*	*0.01*
	CG-207	−0.14	1	0.09	1	−0.14	0.5
	*CG-137*	*−0.34*	*5.10* ^*−3*^	*−0.28*	*0.11*	*−0.32*	*10* ^*−4*^
	*CG-108*	*−0.29*	*0.08*	*−0.28*	*0.05*	*−0.30*	*4.10* ^*−4*^
	Average*	*−0.30*	*0.017*	*−0.25*	*0.17*	*−0.29*	*6.10* ^*−4*^
	CG-77	−0.23	0.25	−0.09	1	−0.17	0.3
	CG + 97	−0.22	0.29	−0.04	1	−0.12	1

To account for multiple CG testing, we used Bonferroni correction of the *P* values studied. *P* values greater than 1 are equated to 1. *Values for the averaged 6 CGs from −108 to −232.

Italicized letters and numbers indicate significant correlation.

### Relationship between methylation and genetic variation at the IGF1 locus

**Figure 4:**
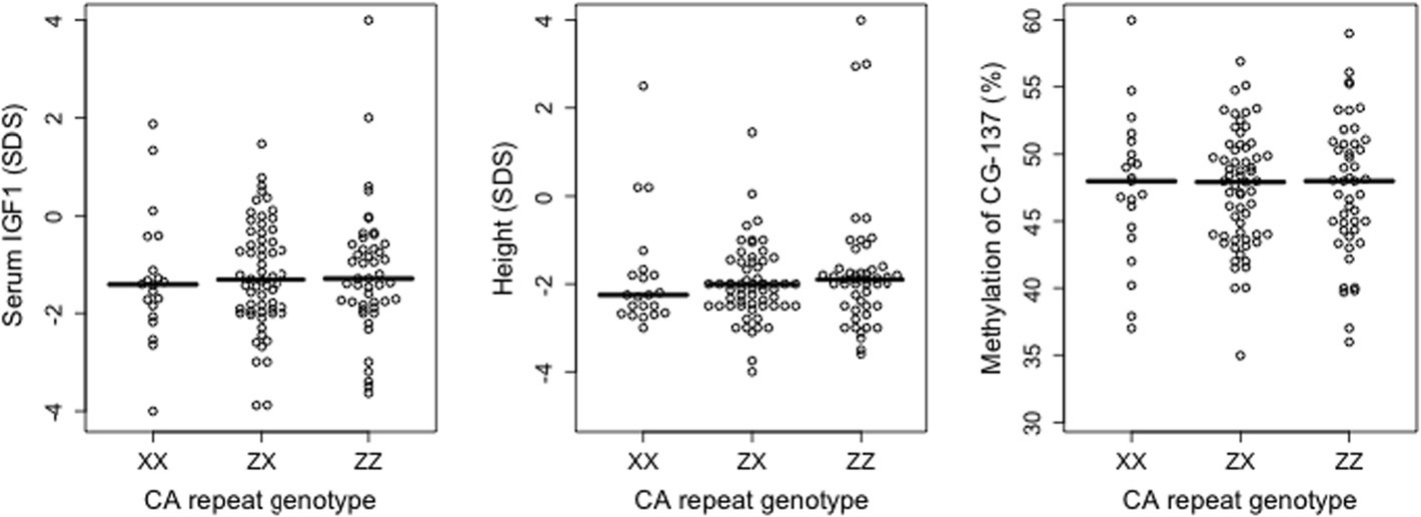
**Lack of relationship between the genotype categories of the microsatellite polymorphism upstream to the** ***IGF1*** **gene and height, circulating IGF1 or CG-137 methylation (** ***N*** **= 130).**

We found no relationship between the common CA repeat variant located −822 bp from the P2 transcription start site (TSS) and methylation of P1 or P2 promoters (Figure 4) or between this repeat variant and height or circulating IGF1 (Figure 4).

### Relationship between methylation and IGF1 transcripts in children PBMC

**Figure 5:**
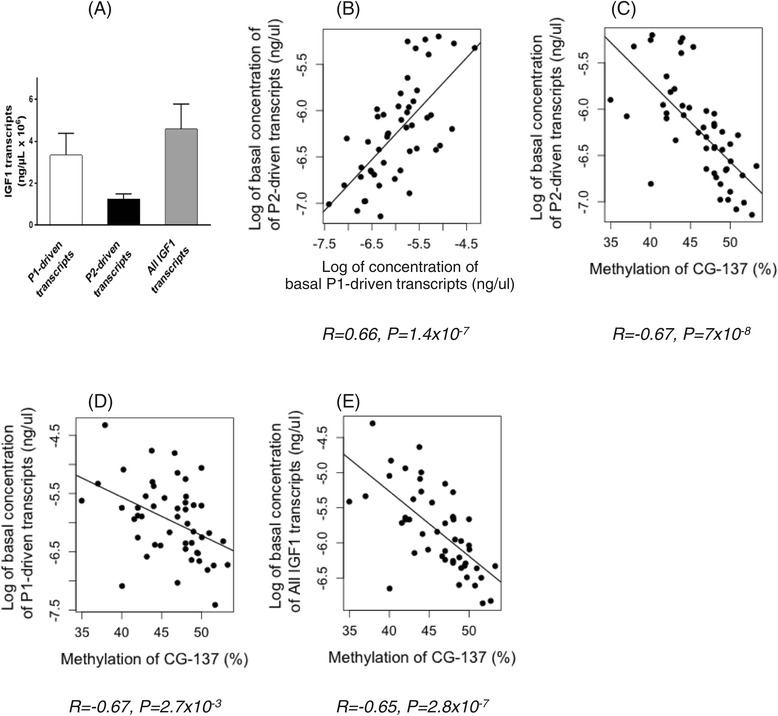
***IGF1*** **transcripts. (A)** P1-driven, P2-driven, and total *IGF1* transcripts in the PBMC from 49/216 children. **(B)** Correlation between class I P1-driven transcripts and class II P2-driven transcripts *Y* = 0.55X − 2.96 (*R* = 0.66, *P* = 1.4 × 10^−7^). **(C)** Inverse correlation between CG-137 methylation and P2-driven transcripts *Y* = −0.09X − 2.3 (*R* = −0.67, *P* = 7 × 10^−8^). (**D**) Inverse correlation between CG-137 methylation and P1-driven transcripts *Y* = −0.06X − 2.93 (*R* = −0.67, *P* = 2.7 × 10^−3^). **(E)** Inverse correlation between CG-137 methylation and all *IGF1* transcripts *Y* = −0.09X − 1.6 (*R* = −0.65, *P* = 2.8 × 10^−7^).

Class I transcripts accounted for 73% of the total *IGF1* transcripts and class II for the remaining 27% in the PBMC of the studied children (Figure 5A). Within an individual, the levels of class I and class II transcripts were highly correlated (*R* = 0.66; *P* = 1.4 × 10^−7^) (Figure 5B).

Class II transcripts were inversely correlated with CG-137 methylation (*R* = −0.67; *P* = 7 × 10^−8^) (Figure 5C), which accounted for 45% of the variation in class II transcripts in PBMC. CG-137 methylation showed only a weak inverse correlation with class I transcripts (Figure 5D) and a highly significant correlation with total *IGF1* transcripts (Figure 5E).

### Artificial demethylation of P2 promoter increases transcription in a reporter gene assay

**Figure 6:**
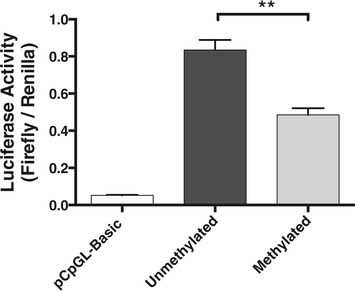
.**The demethylation of the** ***IGF1*** **-P2 region increases luciferase activity.** Results of luciferase assays in transiently transfected HEK293 cells. Forty-eight hours after transfection, basal activity of unmethylated and methylated, Firefly luciferase reporter plasmids containing *IGF1* promoter 2 CG fragment, and empty Firefly luciferase reporter plasmid, pCpGL-Basic, was measured, and normalized to the activity of co-transfected Renilla luciferase plasmid. Results were analyzed by paired *t*-test, **P < 10^−4^, and bars represent three independent experiments (mean ± SEM).

To test *in vitro* whether the methylation of the P2 promoter affects *IGF1* gene expression, we used a reporter gene construct made by inserting a fragment of *IGF1* P2 promoter extending from −1,014 to +64 bp of the TSS - thus containing 7 of the studied CGs - into a promoter-less CpG-free luciferase expression plasmid. Constructed plasmid was mock-methylated or methylated with M.SssI CpG methyltransferase, then transiently transfected into HEK 293 cells in which basal luciferase gene expression was measured. Demethylation of the human *IGF1* P2 promoter increased luciferase reporter gene expression by 57% (Figure 6), indicating that the methylation status of the P2 promoter is a significant regulator of transcriptional activity of this promoter in a plasmid environment devoid of chromatin.

## Discussion

Our observations indicate that the methylation of the P2 promoter of *IGF1* in PBMC is strongly and negatively associated with serum IGF1 and child growth. The P2 promoter can thus be considered an epigenetic quantitative trait locus (QTL^epi^) [13] for these traits. To our knowledge, QTL^epi^ have yet been identified only in plants [12,13]. It is remarkable that P2 promoter methylation contributes 13% to individual height variance, a much greater contribution than ‘major’ genetic variants that account for less than 0.3% each [10,11,20,38-40]. This underscores the potential quantitative importance of epigenetics in the variation of phenotypes.

The molecular causes of idiopathic short stature are multiple [2], as for any multifactorial trait influenced by individual genotypes and environmental factors. High levels of CG methylation at the P2 promoter now appear as one of the many molecular mechanisms responsible for ‘idiopathic’ short stature.

Another potential application of our observation is the secular increase of height [41]. One could speculate that *IGF1* P2 promoter methylation might be an epigenetic link between genetic and environmental determinants of height and contribute to the secular increase in growth observed in genetically stable populations. Indeed, modifications of DNA methylation patterns can be inherited trans-generationally, through incomplete erasure of epigenetic patterning in the germline. An accumulation of epigenetic changes through generations would then provide a valuable, reversible mechanism of adaptation to progressively changing environments. In this respect, the known interaction between energy metabolism, body composition, and IGF1 physiology [42] may stimulate the research of epigenetic links between evolving nutritional factors and height changes throughout generations.

The mechanisms generating variation of CG methylation across humans are not known [43], and this lack of knowledge applies to the individual variations observed in the CG methylation of the *IGF1* P2 promoter. Variation in methylation can arise through *cis* [44-46] or *trans* genetic effects [47,48] or as a result of stochastic events, developmental changes, or exposure to environmental cues [49,50]. The variation in DNA methylation patterns observed in MZ twins suggests that maternal environment affects the fetal epigenome [49,51].

The inverse correlation of P2 promoter methylation with both height and circulating IGF1 and the strong positive correlation between serum IGF1 and height suggest that the epigenetic effects of P2 methylation on child growth are mediated by primary effects on IGF1 production. The *IGF1* gene is expressed in most, if not all, tissues of the body, but the liver and the chondrocytes of the growth plates are the main regulators of systemic IGF1 production and skeletal growth, respectively. Mice models have revealed that GH effects on skeletal growth are mediated by IGF1 produced *in situ* by chondrocytes, not by circulating IGF1 produced by the liver [52,53]. The close correlation of P2 methylation with circulating IGF1 and height supports that the correlation between P2 methylation and *IGF1* gene expression observed in PBMC has a biological relevance to IGF1 and growth physiology. However, our observation should be interpreted with caution because of the lack of data in growth-related tissues. Given the tissue specificity of cell epigenomes, the lack of analysis of physiological tissues and the use of blood cells as surrogates [54,55] are common but major weaknesses of epigenetic epidemiology [14,56]. Measurement of CG methylation in liver and growth plates in a limited number of children undergoing surgical procedures showed that methylation was much lower in these tissues than in blood cells, indicating that the P2 and P1 promoters are tissue-specific differentially methylated regions (t-DMR). One further caveat of using DNA extracted from WBC is the relative contributions made by the respective cell types [57], although the overall impact of blood cell composition across individuals is not considered to be substantial [55]. Herein, CG methylation levels were comparable in WBC, PBMC, and CD4^+^ T lymphocytes.

The molecular mechanisms that link CG methylation of the P2 promoter and *IGF1* gene expression are unknown. In general, gene expression is regulated epigenetically by DNA methylation, histone modifications, and nucleosome positioning. The P2 promoter of *IGF1* belongs to the category of non-CG island promoters, more precisely to the family of low CG promoters, where methylation is known to contribute to the regulation of gene expression [58]. Despite the fact that 45% of all human gene promoters, particularly those controlling the expression of tissue-specific genes, do not lie within CG islands [59], little is known about their regulation and the potential role of methylation as a transcriptional control mechanism [60]. Among the few studies that have investigated the correlation between DNA methylation and expression of genes having non-CG island promoters, the majority do not support the view that DNA methylation directly leads to transcription silencing of these genes [60]. A number of genes with non-CG island promoters display a tissue-specific methylation pattern, suggesting that CG methylation may play a role in the establishment and maintenance of tissue-specific expression patterns [61]. Some studies have shown that there is an inverse correlation between methylation and gene expression [61], whereas other studies have reported that CG-poor promoters could be still expressed when they are methylated [62]. However, when promoters were categorized into low, intermediate, and high CG density, it was found that the inverse correlation between methylation and expression holds in promoters with low CG density [63] and we suspect this could apply to the CG-poor P2 promoter of the *IGF1* gene.

To assess whether DNA methylation can directly regulate the P2 promoter, we utilized a CG-less luciferase-gene containing vector to perform the luciferase reporter assay. This CG-less vector overcomes the problems previously associated with testing methylation-sensitive promoter activity *in vitro*, notably the existence of CG sites in the coding region of the luciferase gene and vector backbone, which potentially contributes to the repression of the promoter [64]. The Stat5b binding sites being located outside of the transfected P2 promoter [65], Stat5b could not contribute to the observed regulation of gene expression in our transfection experiments. These experiments demonstrate that DNA methylation of the non-CG island P2 promoter of *IGF1* can directly silence gene expression, as previously shown when plasmid constructs containing the non-CG island promoters of *LAMB3* and *RUNX3* genes were transfected into HaCaT cells and 623 melanoma cells [60].

The main physiological regulator of IGF1 is GH. The importance of epigenetic mechanisms in the regulation of *IGF1* gene expression by GH starts to be unraveled in the liver of hypophysectomised rats, where GH induces dramatic changes in chromatin at the *IGF1* locus and activates *IGF1* transcription by distinct promoter-specific epigenetic mechanisms [66,67]. The proximal part of rat P2 is an important site of transcriptional regulation by GH via Stat5b [65]. In the rat liver, GH induces rapid and dramatic changes in chromatin at the P2 promoter and activates *IGF1* transcription by specific epigenetic mechanisms [66,67]. At promoter P2, GH facilitates recruitment then activation of RNA Pol II to initiate transcription, whereas at promoter P1, GH causes RNA Pol II to be released from a previously recruited poised and paused pre-initiation complex [66]. CG locations are different in human and rat *IGF1* promoters, and the pattern of methylation or transcriptional effects of these CGs are unknown in rats. Taken together, our observations in human PBMC and transfected cells support that P2 promoter methylation is a strong negative and direct modulator of P2-driven transcription of the human *IGF1* gene, through mechanisms that do not involve Stat5b intervention.

Many questions are left unanswered by the present study, including the epigenetic participation of the P2 promoter to the ‘missing heritability/causality’ of human height variability [68,69] and to the phenotypic plasticity of growth physiology [70]. Another potential application of our finding comes from the pleiotropic role of IGF1 in many developmental and physiological or pathological processes related to aging, longevity, energy metabolism, brain functions, and cancer.

## Conclusions

The P2 promoter of the *IGF1* gene is a significant QTL^epi^ for skeletal growth and serum IGF1. The relationship between P2 methylation and these growth-related traits seems to be mediated by primary transcriptional effects taking place at the P2 promoter.

## Methods

### Participants

To explore the relation between *IGF1* promoter methylation and height, we recruited children who have not yet entered puberty. Mean age was 9.7 years in boys and 9.6 in girls. Testosterone levels were lower than 0.1 ng/ml in all boys, and no girl had any breast development at time of study. This criterion of selection avoids the confounding effect of the variable tempo of sexual maturation, which adds to the variability of adolescent growth, adult height, and serum IGF1. Our discovery cohort was formed with 110 Caucasian white children (58 M/52 F) who had been recruited by the EpiGrowth Consortium. They either had a history of short stature or were control children of various statures. For our replication cohort, we used 106 unrelated healthy white children (63 M/43 F) of the GH-Pharmacogenetic Cohort, which include children with short stature [71]. Children were all healthy, had normal clinical examination and no signs of puberty. GH deficiency was excluded with a stimulated GH peak >15 ng/ml. All had normal TSH levels; subtle chondrodysplasia were excluded by forearm, pelvis, and spine radiographs.

Trained nurses performed height measurements in duplicate using the Harpenden stadiometer. Ten milliliters of peripheral blood samples was obtained, from which WBC and/or PBMC or CD4^+^ T lymphocytes were purified immediately. WBC and PBMC were counted at time of sampling. Samples were obtained from liver and growth plates of children undergoing surgical procedures for reasons independent from the current study.

Parents gave written informed consent for the current study and for using surgical specimens, according to the French rules of bioethics in biomedical research. The research protocol received the agreement of our Institutional Review Board of Paris Sud University.

### Serum IGF1 measurements

Serum IGF1 concentration was measured around 7.00 to 8.00 am before breakfast in 184/216 children using an immune-radiometric assay after ethanol-acid extraction using DSL-5600 Active (Diagnostic System Laboratories, Webster, TX, USA) or Cisbio reagents (Cisbio International, Codolet, France*)*. Intra- and inter-series coefficients of variation were 1.5% and 3.7% at 260 ng/ml and 3.9% at 760 ng/ml. The sensitivity was 4 ng/ml. IGF1 SDS were calculated using the norms of Alberti *et al*. in French children [72].

### Pyrosequencing-based bisulfite PCR analysis

For promoter P1, we studied 9 CGs located over a 800-bp distance, the closest CG being 225 bp upstream from the corresponding major TSS [73]. For promoter P2, we studied 7/8 CGs located upstream from the major TSS within the proximal part of the promoter and 1 CG located 97 bp downstream this TSS. CGs are denominated according to their position versus each promoter TSS (Figure 1). The methylation of CG-22 could not be measured for technical reasons. Nucleic acids were extracted from WBC or PBMC using Gentra Puregene blood kit (Qiagen, Hilden, Germany). In a subset of 20 patients, WBC and PBMC were measured in the same samples. Methylation was measured in other patients using PBMC. A bisulfite-PCR-pyrosequencing technique [74] was used to measure the methylation of the CGs. We improved the resolution of this method from a handful of bases to up to 100 nucleotides, with the ability to quantify methylation in the same sample of blood with a coefficient of variation (SD/mean) as little as 1% to 5%. Briefly, 400 nanograms of genomic DNA were treated with EZ DNA Methylation-Gold Kit, according to manufacturer’s protocol (Zymo Research Corporation, Irvine, CA, USA). The bisulfite-treated genomic DNA was PCR-amplified using unbiased *IGF1* primers (see Additional file 1: Table S1) and performed quantitative pyrosequencing using a PyroMark Q96 ID Pyrosequencing instrument (Qiagen, Hilden, Germany). Pyrosequencing assays were designed using MethPrimer (http://www.urogene.org/methprimer/index1.html). Biotin-labeled single-stranded amplicons were isolated according to protocol using the Qiagen Pyromark Q96 Work Station (Qiagen, Hilden, Germany) and underwent pyrosequencing with 0.5 μM primer. The percent methylation for each of the CGs within the target sequence was calculated using PyroQ CpG Software (Qiagen, Hilden, Germany).

Liver and tibial growth plate samples were washed with PBS and dissected to remove conjunctive tissue and blood. The biopsies were ground in cell lysis solution (Qiagen, Hilden, Germany) with Minilys® (Bertin Technologies, Montigny-le-Bretonneux) according to manufacter’s recommendation. Nucleic acids were extracted using Gentra Puregene tissue kit (Qiagen, Hilden, Germany).

### Study of *IGF1* transcripts in PBMC

PBMC were almost immediately isolated from fresh blood in 49 children using a density gradient. Four milliliters of fresh blood was mixed with 4 ml of NaCl 154 mM, and then, 4 ml of Lymphoprep solution (Eurobio, Paris, France) was added to diluted blood and centrifuged for 20 min at room temperature at 800*g*. After centrifugation, the interphase containing PBMC was carefully aspirated and the cells were mixed with NaCl. The cell suspension was centrifuged at 300*g*, and the cell pellet washed with PBS before 700 μl of Qiazol (Qiagen, Hilden, Germany) was added and the pellet frozen in −80°C. Total RNA was isolated using miRNA mini kit (Qiagen), according to manufacturer’s protocol. Genomic DNA was removed using DNAse treatment. RNA integrity was assessed by bioanalyser (Agilent 2100, Agilent Technologies, Palo Alto, CA, USA) and expressed as RNA integrity number (RIN) considered acceptable within the range of 7 to 10. One microgram RNA was reverse-transcribed with random hexamers and oligo(dT) in a final volume of 18 μl using PrimeScript RT reagent kit (TAKARA, Tokyo, Japan). Parallel reactions without reverse transcriptase enzyme were prepared as negative controls.

qPCR for samples were prepared in triplicate and run on ABI 7500 fast (Applied Biosystems, Foster City, CA, USA). We amplified P1-driven, P2-driven, and whole transcripts of *IGF1* transcripts using predesigned Custom TaqMan gene expression assay (Integrated DNA Technologies IDT, Coralville, IA, USA) (Additional file 1: Table S1). To detect P1-driven transcripts, P2-driven transcripts, and global *IGF1* transcripts separately, TaqMan assays were derived respectively from exons 1 to 3, exons 2 to 3, and exons 3 to 4. We measured P1-driven transcripts, P2-driven transcripts, and global transcripts of *IGF1* gene in PBMC using absolute quantification with an external calibration curve model.

A calibration standard curve was established using a cDNA clone containing qPCR product targeted by each *IGF1* TaqMan assay. A series of seven concentrations of *IGF1* cDNA clone was prepared by serial dilution. The standard curves were constructed by plotting the cycle threshold *vs.* the concentration of DNA (log_10_ scale). The equation, slope, and correlation coefficient for each curve are presented in Additional file 2: Figure S1.

### Construction of the reporter gene plasmid

Fragment of human *IGF1*-P2, −1,014 to +64, including CGs located within the proximal part of the promoter 2, was PCR amplified from the human genomic DNA using SpeI restriction site added forward primer (Additional file 1: Table S1) and NcoI restriction site added reverse primer with Phusion High-Fidelity DNA Polymerase (Thermo Fisher Scientific, Waltham, MA, USA). PCR product cloned into upstream of the firefly luciferase gene in the promoter-less CpG-free pCpGL-Basic vector (gift from Rehli’s Lab, Regensburg, Germany) between SpeI-NcoI sites and transformed into PIR1 competent cells (Invitrogen, Carlsbad, CA, USA) for plasmid production.

*In vitro* methylation protocol was adapted from Klug and Rehli [64]. *IGF1*-P2, −1,014 to +64, in pCpGL-Basic was incubated at 37°C for 6 h with M.SssI CpG Methyltransferase (New England Biolabs, Ipswich, MA, USA) (2.5 U/μg DNA), in the presence of a methyl group donor 160 μM S-adenosylmethionine (SAM). One hundred sixty micromolar SAM was added every 2 h during incubation. Unmethylated plasmid was treated as above but without the M.SssI CpG Methyltransferase. Plasmid DNA was phenol/chloroform extracted and ethanol precipitated and quantified using a NanoDrop Spectrophotometer (ND-1000, NanoDrop Technologies, Wilmington, DE, USA). The completeness of the methylation for both methylated and unmethylated plasmids were first confirmed with methylation-sensitive restriction enzyme HpaII and methylation-insensitive (MspI) restriction enzyme.

### Cell culture, transient transfection, and luciferase reporter assays

Human embryonic kidney 293 (HEK293) cell line (American Type Culture Collection (ATCC)) was maintained in Dulbecco’s Modified Eagle’s medium (DMEM; Life Technologies, Carlsbad, CA, USA) supplemented with 10% fetal bovine serum (HyClone, Thermo Fisher Scientific, Waltham, MA, USA), penicillin/streptomycin, and glutamine (PAA Laboratories, Pashing, Austria). Cells were maintained at 37°C in a humidified atmosphere containing 95% air and 5% CO2. HEK293 cells in 96-well plates were co-transfected using X-tremeGENE HP DNA Transfection Reagent (Roche, Indianapolis, IN, USA) with 80 ng/well methylated or unmethylated promoter-less CpG-free *Firefly* Luciferase expressing plasmid containing *IGF1*-P2 fragment with the 8 studied CGs, and 1 ng/well pRL-TK expressing plasmid (Promega, Madison, WI, USA), containing a constitutively expressed *Renilla* luciferase reporter gene used as an internal control for transfection efficiency. Luminescence was measured 48 h after transfection using dual-luciferase reporter assay system (Promega, Madison, WI, USA), and Centro LB 960 luminometer (Berthold Technologies, Oak Ridge, TN, USA). Results from three independent transfection experiments were averaged for comparison.

### Genotyping distribution of (CA)n *IGF1* repeat

Genotyping of the *IGF1* gene repeats, located 1 kb upsteam from the major P1 transcription start site, was adapted from Cleveland *et al*. [75] and Arends *et al*. [76] protocols. PCR were carried out in 25-μl volumes with 30 ng of genomic DNA using 2 μM of each primer (see Additional file 1: Table S1), 100 μM dNTPs, 1.5 mM MgCl_2_ 25 mM, 2.5 μl of the manufacturer’s standard buffer and 1U of Taq RED polymerase (Eurogentec, Angers). Samples were processed through one cycle of 3 min at 94°C, 35 temperature cycles consisting of 30 s at 94°C (denaturation), 30 s at 55°C (annealing), and 30 s at 72°C (elongation), with the last elongation step lengthened to 10 min. The forward primer was 5′labeled with FAM fluorescent dye for automated fragment analysis (ABI Applied Biosystems, Foster City, CA, USA). The PCR amplified products were diluted 1/10, and 1 μl of the diluted PCR product is added to 10 μl of formamide mix and 0.5 μl of Gen Scan 500 marquer. GeneMapper 4 was used to analyzed the length of samples.

### Calculations and statistical methods

IGF1 levels and height were expressed as SDS to adjust for age and sex. Pearson correlations were calculated as adjusted *R*-squared. The fraction of explained variance across children was calculated under the linear regression model, using the usual definition: *r*^2^ × 100. Wilcoxon rank tests and unpaired Student’s *t*-tests were both used to compare methylation levels in studied tissues and the results of transfections with the demethylated or methylated P2 promoter. The Bonferroni correction was used to account for the number of CG tested. All statistics were computed using R 2.10.1. The results are expressed as mean ± SD.

## Additional files

Additional file 1:
**List of primers used in our study. Sequences are given from 5to 3′.**
Additional file 2:
**Standard curves used for the measurement of** ***IGF1*** **gene transcript content in PBMC.**

## Abbreviations

IGF1: insulin-like growth factor 1
PBMC: peripheral blood mononuclear cells
QTL: quantitative trait locus
QTL^epi^: epigenetic quantitative trait locus
WBC: whole blood cells.

## References

[1] Cohen P, Rogol AD, Deal CL, Saenger P, Reiter EO, Ross JL (2007). Consensus statement on the diagnosis and treatment of children with idiopathic short stature: a summary of the Growth Hormone Research Society, the Lawson Wilkins Pediatric Endocrine Society, and the European Society for Paediatric Endocrinology Workshop. J Clin Endocrinol Metab.

[2] Rosenfeld RG (2005). The molecular basis of idiopathic short stature. Growth Horm IGF Res.

[3] Cole TJ (2003). The secular trend in human physical growth: a biological view. Econ Hum Biol.

[4] Galton F, Dickson JDH (1886). Family likeness in stature. Proc R Soc Lond.

[5] Visscher PM, McEvoy B, Yang J (2010). From Galton to GWAS: quantitative genetics of human height. Genet Res.

[6] Hirschhorn JN, Gajdos ZKZ (2011). Genome-wide association studies: results from the first few years and potential implications for clinical medicine. Annu Rev Med.

[7] Fisher RA (1919). XV. - The correlation between relatives on the supposition of Mendelian inheritance. Earth Environ Sci Trans R Soc Edinb.

[8] Silventoinen K, Sammalisto S, Perola M, Boomsma DI, Cornes BK, Davis C (2003). Heritability of adult body height: a comparative study of twin cohorts in eight countries. Twin Res.

[9] Wood AR, Esko T, Yang J, Vedantam S, Pers TH, Gustafsson S (2014). Defining the role of common variation in the genomic and biological architecture of adult human height. Nat Genet.

[10] Lango Allen H, Estrada K, Lettre G, Berndt SI, Weedon MN, Rivadeneira F (2010). Hundreds of variants clustered in genomic loci and biological pathways affect human height. Nature.

[11] Yang J, Benyamin B, McEvoy BP, Gordon S, Henders AK, Nyholt DR (2010). Common SNPs explain a large proportion of the heritability for human height. Nat Genet.

[12] Murrell A, Rakyan VK, Beck S (2005). From genome to epigenome. Hum Mol Genet.

[13] Johannes F, Colot V, Jansen RC (2008). Epigenome dynamics: a quantitative genetics perspective. Nat Rev Genet.

[14] Rakyan VK, Down TA, Balding DJ, Beck S (2011). Epigenome-wide association studies for common human diseases. Nat Rev Genet.

[15] Woods KA, Camacho-Hübner C, Savage MO, Clark AJ (1996). Intrauterine growth retardation and postnatal growth failure associated with deletion of the insulin-like growth factor I gene. N Engl J Med.

[16] Lupu F, Terwilliger JD, Lee K, Segre GV, Efstratiadis A (2001). Roles of growth hormone and insulin-like growth factor 1 in mouse postnatal growth. Dev Biol.

[17] Rogers I, Metcalfe C, Gunnell D, Emmett P, Dunger D, Holly J (2006). Insulin-like growth factor-I and growth in height, leg length, and trunk length between ages 5 and 10 years. J Clin Endocrinol Metab.

[18] Cohen P (2006). Controversy in clinical endocrinology: problems with reclassification of insulin-like growth factor I production and action disorders. J Clin Endocrinol Metab.

[19] Frayling TM, Hattersley AT, McCarthy A, Holly J, Mitchell SMS, Gloyn AL (2002). A putative functional polymorphism in the IGF-I gene association studies with type 2 diabetes, adult height, glucose tolerance, and fetal growth in U.K. Populations. Diabetes.

[20] Lettre G, Butler JL, Ardlie KG, Hirschhorn JN (2007). Common genetic variation in eight genes of the GH/IGF1 axis does not contribute to adult height variation. Hum Genet.

[21] Kim J-J, Lee H-I, Park T, Kim K, Lee J-E, Cho NH (2009). Identification of 15 loci influencing height in a Korean population. J Hum Genet.

[22] Okada Y, Kamatani Y, Takahashi A, Matsuda K, Hosono N, Ohmiya H (2010). A genome-wide association study in 19 633 Japanese subjects identified LHX3-QSOX2 and IGF1 as adult height loci. Hum Mol Genet.

[23] Harrela M, Koistinen H, Kaprio J, Lehtovirta M, Tuomilehto J, Eriksson J (1996). Genetic and environmental components of interindividual variation in circulating levels of IGF-I, IGF-II, IGFBP-1, and IGFBP-3. J Clin Invest.

[24] Kao PC, Matheny AP, Lang CA (1994). Insulin-like growth factor-I comparisons in healthy twin children. J Clin Endocrinol Metab.

[25] Verhaeghe J, Loos R, Vlietinck R, Van Herck E, van Bree R, DSchutter AM (1996). C-peptide, insulin-like growth factors I and II, and insulin-like growth factor binding protein-1 in cord serum of twins: genetic versus environmental regulation. Am J Obstet Gynecol.

[26] Palles C, Johnson N, Coupland B, Taylor C, Carvajal J, Holly J (2008). Identification of genetic variants that influence circulating IGF1 levels: a targeted search strategy. Hum Mol Genet.

[27] Al-Zahrani A, Sandhu MS, Luben RN, Thompson D, Baynes C, Pooley KA (2006). IGF1 and IGFBP3 tagging polymorphisms are associated with circulating levels of IGF1, IGFBP3 and risk of breast cancer. Hum Mol Genet.

[28] Wong H-L, DeLellis K, Probst-Hensch N, Koh W-P, Berg DVD, Lee H-P (2005). A new single nucleotide polymorphism in the insulin-like growth factor I regulatory region associates with colorectal cancer risk in Singapore Chinese. Cancer Epidemiol Biomarkers Prev.

[29] Ester WA, Hokken-Koelega ACS (2008). Polymorphisms in the IGF1 and IGF1R genes and children born small for gestational age: results of large population studies. Best Pract Res Clin Endocrinol Metab.

[30] Vaessen N, Janssen JA, Heutink P, Hofman A, Lamberts SWJ, Oostra BA (2002). Association between genetic variation in the gene for insulin-like growth factor-I and low birthweight. Lancet.

[31] Rotwein P (2012). Mapping the growth hormone - Stat5b - IGF-I transcriptional circuit. Trends Endocrinol Metab.

[32] Bock C, Walter J, Paulsen M, Lengauer T (2008). Inter-individual variation of DNA methylation and its implications for large-scale epigenome mapping. Nucleic Acids Res.

[33] Kuroda A, Rauch TA, Todorov I, Ku HT, Al-Abdullah IH, Kandeel F (2009). Insulin gene expression is regulated by DNA methylation. PLoS ONE.

[34] Murgatroyd C, Patchev AV, Wu Y, Micale V, Bockmühl Y, Fischer D (2009). Dynamic DNA methylation programs persistent adverse effects of early-life stress. Nat Neurosci.

[35] Yang H, Adamo ML, Koval AP, McGuinness MC, Ben-Hur H, Yang Y (1995). Alternative leader sequences in insulin-like growth factor I mRNAs modulate translational efficiency and encode multiple signal peptides. Mol Endocrinol.

[36] Adamo ML, Ben-Hur H, Roberts CT, LeRoith D (1991). Regulation of start site usage in the leader exons of the rat insulin-like growth factor-I gene by development, fasting, and diabetes. Mol Endocrinol.

[37] Oberbauer AM (2013). The regulation of IGF-1 gene transcription and splicing during development and aging. Front Endocrinol.

[38] Weedon MN, Lettre G, Freathy RM, Lindgren CM, Voight BF, Perry JRB (2007). A common variant of HMGA2 is associated with adult and childhood height in the general population. Nat Genet.

[39] Sanna S, Jackson AU, Nagaraja R, Willer CJ, Chen W-M, Bonnycastle LL (2008). Common variants in the GDF5-UQCC region are associated with variation in human height. Nat Genet.

[40] Weedon MN, Lango H, Lindgren CM, Wallace C, Evans DM, Mangino M (2008). Genome-wide association analysis identifies 20 loci that influence adult height. Nat Genet.

[41] Bogin B (2013). Secular changes in childhood, adolescent and adult stature. Nestlé Nutr Inst Workshop Ser.

[42] Clemmons DR, Underwood LE (1991). Nutritional regulation of IGF-I and IGF binding proteins. Annu Rev Nutr.

[43] Bird A (2011). Putting the DNA back into DNA methylation. Nat Genet.

[44] Knight JC (2004). Allele-specific gene expression uncovered. Trends Genet TIG.

[45] Meaburn EL, Schalkwyk LC, Mill J (2010). Allele-specific methylation in the human genome: implications for genetic studies of complex disease. Epigenetics.

[46] Fradin D, Le Fur S, Mille C, Naoui N, Groves C, Zelenika D (2012). Association of the CpG methylation pattern of the proximal insulin gene promoter with type 1 diabetes. PLoS ONE.

[47] Richards EJ (2008). Population epigenetics. Curr Opin Genet Dev.

[48] Bell JT, Pai AA, Pickrell JK, Gaffney DJ, Pique-Regi R, Degner JF (2011). DNA methylation patterns associate with genetic and gene expression variation in HapMap cell lines. Genome Biol.

[49] Fraga MF, Ballestar E, Paz MF, Ropero S, Setien F, Ballestar ML (2005). Epigenetic differences arise during the lifetime of monozygotic twins. Proc Natl Acad Sci U S A.

[50] Kaminsky ZA, Tang T, Wang S-C, Ptak C, Oh GHT, Wong AHC (2009). DNA methylation profiles in monozygotic and dizygotic twins. Nat Genet.

[51] Petronis A (2006). Epigenetics and twins: three variations on the theme. Trends Genet TIG.

[52] Yakar S, Pennisi P, Wu Y, Zhao H, LeRoith D, Cianfarani S, Clemmons DR, Savage MO (2005). Clinical relevance of systemic and local IGF-I. Endocrine Development.

[53] Liu JL, Yakar S, LeRoith D (2000). Conditional knockout of mouse insulin-like growth factor-1 gene using the Cre/loxP system. Proc Soc Exp Biol Med.

[54] Feinberg AP, Irizarry RA, Fradin D, Aryee MJ, Murakami P, Aspelund T (2010). Personalized epigenomic signatures that are stable over time and covary with body mass index. Sci Transl Med.

[55] Talens RP, Boomsma DI, Tobi EW, Kremer D, Jukema JW, Willemsen G (2010). Variation, patterns, and temporal stability of DNA methylation: considerations for epigenetic epidemiology. FASEB J.

[56] Mill J, Heijmans BT (2013). From promises to practical strategies in epigenetic epidemiology. Nat Rev Genet.

[57] Wu H-C, Delgado-Cruzata L, Flom JD, Kappil M, Ferris JS, Liao Y (2011). Global methylation profiles in DNA from different blood cell types. Epigenetics.

[58] Xie W, Schultz MD, Lister R, Hou Z, Rajagopal N, Ray P (2013). Epigenomic analysis of multilineage differentiation of human embryonic stem cells. Cell.

[59] Takai D, Jones PA (2002). Comprehensive analysis of CpG islands in human chromosomes 21 and 22. Proc Natl Acad Sci U S A.

[60] Han H, Cortez CC, Yang X, Nichols PW, Jones PA, Liang G (2011). DNA methylation directly silences genes with non-CpG island promoters and establishes a nucleosome occupied promoter. Hum Mol Genet.

[61] Eckhardt F, Lewin J, Cortese R, Rakyan VK, Attwood J, Burger M (2006). DNA methylation profiling of human chromosomes 6, 20 and 22. Nat Genet.

[62] Weber M, Hellmann I, Stadler MB, Ramos L, Pääbo S, Rebhan M (2007). Distribution, silencing potential and evolutionary impact of promoter DNA methylation in the human genome. Nat Genet.

[63] Gal-Yam EN, Egger G, Iniguez L, Holster H, Einarsson S, Zhang X (2008). Frequent switching of polycomb repressive marks and DNA hypermethylation in the PC3 prostate cancer cell line. Proc Natl Acad Sci U S A.

[64] Klug M, Rehli M (2006). Functional analysis of promoter CpG methylation using a CpG-free luciferase reporter vector. Epigenetics.

[65] Varco-Merth B, Mirza K, Alzhanov DT, Chia DJ, Rotwein P (2012). Biochemical characterization of diverse Stat5b-binding enhancers that mediate growth hormone-activated insulin-like growth factor-I gene transcription. PLoS ONE.

[66] Chia DJ, Young JJ, Mertens AR, Rotwein P (2010). Distinct alterations in chromatin organization of the two IGF-I promoters precede growth hormone-induced activation of IGF-I gene transcription. Mol Endocrinol.

[67] Chia DJ, Rotwein P (2010). Defining the epigenetic actions of growth hormone: acute chromatin changes accompany GH-activated gene transcription. Mol Endocrinol.

[68] Slatkin M (2009). Epigenetic inheritance and the missing heritability problem. Genetics.

[69] Manolio TA, Collins FS, Cox NJ, Goldstein DB, Hindorff LA, Hunter DJ (2009). Finding the missing heritability of complex diseases. Nature.

[70] Atchley WR, Zhu J (1997). Developmental quantitative genetics, conditional epigenetic variability and growth in mice. Genetics.

[71] Dos Santos C, Essioux L, Teinturier C, Tauber M, Goffin V, Bougnères P (2004). A common polymorphism of the growth hormone receptor is associated with increased responsiveness to growth hormone. Nat Genet.

[72] Alberti C, Chevenne D, Mercat I, Josserand E, Armoogum-Boizeau P, Tichet J (2011). Serum concentrations of insulin-like growth factor (IGF)-1 and IGF binding protein-3 (IGFBP-3), IGF-1/IGFBP-3 ratio, and markers of bone turnover: reference values for French children and adolescents and z-score comparability with other references. Clin Chem.

[73] Jansen E, Steenbergh PH, LeRoith D, Roberts CT, Sussenbach JS (1991). Identification of multiple transcription start sites in the human insulin-like growth factor-I gene. Mol Cell Endocrinol.

[74] Tost J, Gut IG (2007). DNA methylation analysis by pyrosequencing. Nat Protoc.

[75] Cleveland RJ, Gammon MD, Edmiston SN, Teitelbaum SL, Britton JA, Terry MB (2006). IGF1 CA repeat polymorphisms, lifestyle factors and breast cancer risk in the Long Island Breast Cancer Study Project. Carcinogenesis.

[76] Arends N, Johnston L, Hokken-Koelega A, van Duijn C, de Ridder M, Savage M (2002). Polymorphism in the IGF-I gene: clinical relevance for short children born small for gestational age (SGA). J Clin Endocrinol Metab.

