# Supplementary material for: The IGF1 P2 promoter is an epigenetic QTL for circulating IGF1 and human growth
Source: Clin Epigenetics. 2015 Mar 13;7(1):22. doi: 10.1186/s13148-015-0062-8 (PMC4363053; doi:10.1186/s13148-015-0062-8)
Supplement: Additional file 1: — List of primers used in our study. Sequences are given from 5to 3′. [file 13148_2015_62_MOESM1_ESM.docx]

| **Additional file 1: Table S1.** List of primers used in our study. Sequences are given from 5’ to 3’. | |
| --- | --- |
| **Region** | **Primers** |
| **Pyrosequencing** |  |
| CGs -1044 to -919 | F- TATTGTTTTATAAAATTAAAGGGAA  R- ATTCTAAATAACACCAACTAACTAAC |
| primary for CGs -631. to -308 | F- TGATAGGTAGTTTAGTAGAAGAATAA  R- AAAACTAAAACACAAAAACATTTTT |
| nested 1 for CGs -631 and -611 | F- GTTTTATTTTAGTTGGGTTTTATAG  R- CTCTACAATTTTAACCCTAAAAT |
| nested 2 for CGs -491 to -308 | F- GTAATTGGGATAAGGGGTTATT  R-AACTTTCTACTAAACATAAAAACACAAAC |
| CG -225 | F-TTTTTAATATTGGTAAGGTGTATTAG  R- TTTCCCTTAAAAAAATATATATTAAT |
| Sequencing CG -1044 | F- AGGGAAATAGGTATAAAT |
| Sequencing CG -960 | F- AGTTGGTTTGGATTATGT |
| Sequencing CG -919 | F- GGGATTTGAATGATATTA |
| Sequencing CGs -631 and -611 | R- CTATCTCATACTTTTTCT |
| Sequencing CG -491 | F- TAATTGGGATAAGGGG |
| Sequencing CG -414 | F- ATTTTAGGGTTAAAATTGTAGAGT |
| Sequencing CG -308 | F- ATATTGGTAAGGTGTATT |
| Sequencing CG -225 | F- GGTAGTATAAGTATTTTA |
| CGs -232 to 108 | F-AATTTGGTTGTTGTTGTTAGTGTAT  R- AATTAAACCCTCAAACAATTAAATC |
| CGs -77 and +97 | F-AATTGTTTGAGGGTTTAATTTATAAGA  R-ACTACTAAATCACATAATATATATACACA |
| Sequencing CGs -232. -224. 218 and -207 | F- TGGTTGTTGTTGTTAGTGTAT |
| Sequencing CG -137 | R-ACCAATAACAACAACTTAA |
| Sequencing CG -108 | F- AAGTTGTTGTTATTGGTT |
| Sequencing CG -77 | R- ACTCCCTCAAACCACTTCCTACT |
| Sequencing CG +97 | R- AATTGTTTGAGGGTTTAATTTATAAGA |
| **Quantitative PCR** |  |
| *HPRT1* | Hs.PT.5821454446 Assay IDT |
| Total *IGF1* | Hs.PT.56a.21022358.g IDT |
| P1-driven *IGF1* | N001111285.1.pt.IGF1 IDT |
| P2-driven *IGF1* | N001111284.1.pt.IGF1 IDT |
| **Plasmid** |  |
| Forward | TCAACTAGTATGTGTACTGTTTGCTTCTGCCTAGA |
| Reverse | TCACCATGGTGGCATCTCAGGAACAGCAG |
| **Genotypage *IGF1* CAn** |  |
| Forward | GCTAGCCAGCTGGTGTTATT |
| Reverse | ACCACTCTGGGAGAAGGGTA |

**Additional file 1: Table S2.** CG methylation in the P1 and P2 promoters of the *IGF1* gene. The position of each CG is given as bp from the TSS of the corresponding promoter.

|  |  | **Percent Methylation** | | | |
| --- | --- | --- | --- | --- | --- |
|  |  | **Mean ± SD** | **Extreme Values%** | **Range interval** | **SD/mean** |
| Promoter P1 | CG -1044 | 88 ± 3 | 78 - 95 | 17 | 0.03 |
|  | CG -960 | 79 ± 4 | 75 - 87 | 12 | 0.05 |
|  | CG -919 | 89 ± 6 | 80 - 98 | 18 | 0.07 |
|  | CG -631 | 86 ± 2 | 75 - 91 | 16 | 0.02 |
|  | CG -611 | 92 ± 3 | 85 - 99 | 14 | 0.03 |
|  | CG -491 | 84 ± 4 | 69 - 93 | 24 | 0.05 |
|  | CG -414 | 12 ± 4 | 5 - 36 | 31 | 0.33 |
|  | CG -308 | 10 ± 4 | 3 - 28 | 25 | 0.40 |
|  | CG -225 | 8 ± 2 | 4 - 14 | 10 | 0.25 |
|  |  |  |  |  |  |
|  |  |  |  |  |  |
|  |  |  |  |  |  |
| Promoter  P2 | CG -232 | 66 ± 7 | 46 - 87 | 41 | 0.11 |
|  | CG -224 | 73 ± 7 | 58 - 92 | 34 | 0.10 |
|  | CG -218 | 73 ± 6 | 61 - 89 | 28 | 0.08 |
|  | CG -207 | 44 ± 8 | 20 - 60 | 40 | 0.18 |
|  | CG -137 | 47 ± 4 | 35 - 60 | 25 | 0.09 |
|  | CG -108 | 61 ± 6 | 46 - 74 | 28 | 0.10 |
|  | CG -77 | 48 ± 5 | 35 - 60 | 25 | 0.10 |
|  | CG +97 | 16 ± 3 | 9 - 24 | 15 | 0.19 |

**Additional file 1: Table S3.** CG methylation is not influenced by age. Adult controls from our biobank were used for comparison.

| Age (yrs) | **< 11** | **11-20** | **20-60** | **>60** |
| --- | --- | --- | --- | --- |
| *N* | *185* | *120* | *47* | *46* |
| CG-137 methylation % | 46 ± 5 | 45 ± 5 | 44 ± 4 | 45 ± 4 |

**Additional file 1: Table S4.** Methylation levels in white blood cells (WBC), peripheral blood mononuclear cells (PBMC), CD4^+^T lymphocytes, liver and tibial growth plates.

|  |  | **WBC** | **PBMC** | **CD4^+^T lymphocytes** | **Liver** | **Tibial growth plate** |
| --- | --- | --- | --- | --- | --- | --- |
|  |  | *N=16* | *N=20* | *N=5* | *N=10* | *N=3* |
| Promoter P1 | CG -1044 | 87 ± 2 | 88 ± 4 | 90 ± 2 | 34 ± 5* | 58 ± 3* |
|  | CG -960 | 80 ± 2 | 79 ± 2 | 87 ± 4 | 26 ± 4* | 21 ± 1* |
|  | CG -919 | 90 ± 2 | 88 ± 3 | 96 ± 6 | 27 ± 4* | 34 ± 2* |
|  | CG -631 | 86 ± 2 | 86 ± 2 | 86 ± 1 | 50 ± 15* | 60 ± 7* |
|  | CG -611 | 90 ± 3 | 92 ± 3 | 94 ± 2 | 52 ± 15* | 55 ± 8* |
|  | CG -491 | 83 ± 3 | 84 ± 3 | 85 ± 5 | 46 ± 18* | 59 ± 7* |
|  | CG -414 | 12 ± 3 | 12 ± 2 | 15 ± 5 | 9 ± 6 | 5 ± 2 |
|  | CG -308 | 10 ± 3 | 11 ± 3 | 16 ± 6 | 7 ± 8 | 11 ± 4 |
|  | CG -225 | 8 ± 1 | 8 ± 3 | 10 ± 1 | 7 ± 2 | 7 ± 1 |
|  |  |  |  |  |  |  |
|  |  |  |  |  |  |  |
|  |  |  |  |  |  |  |
| Promoter P2 | CG -232 | 64 ± 4 | 61± 5 | 68 ± 4 | 28 ± 4* | 27 ± 3* |
|  | CG -224 | 68 ± 5 | 73 ± 6 | 72 ± 3 | 29 ± 5* | 28 ± 3* |
|  | CG -218 | 66 ± 5 | 73 ±6 | 74 ± 3 | 30 ± 4* | 31 ± 1* |
|  | CG -207 | 38 ± 7 | 45 ± 7 | 35 ± 3 | 12 ± 2* | 17 ± 1* |
|  | CG -137 | 47 ± 3 | 45 ± 4 | 50 ±4 | 18 ± 4* | 19 ± 2* |
|  | CG -108 | 58 ± 6 | 60 ± 4 | 64 ±3 | 25 ± 6* | 22 ± 2* |
|  | CG -77 | 50 ± 3 | 44 ± 5 | 51 ± 3 | 21 ± 3* | 21 ± 3* |
|  | CG +97 | 18 ± 2 | 17 ± 2 | 18 ± 1 | 9 ± 2* | 10 ± 2* |

**10^-6^<P<0.03 for comparison with CG methylation in PBMC.*
