# Supplementary material for: The IGF1 P2 promoter is an epigenetic QTL for circulating IGF1 and human growth
Source: Clin Epigenetics. 2015 Mar 13;7(1):22. doi: 10.1186/s13148-015-0062-8 (PMC4363053; doi:10.1186/s13148-015-0062-8)
Supplement: Additional file 2: — Standard curves used for the measurement of IGF1 gene transcript content in PBMC. [file 13148_2015_62_MOESM2_ESM.pptx]

## Slide 1
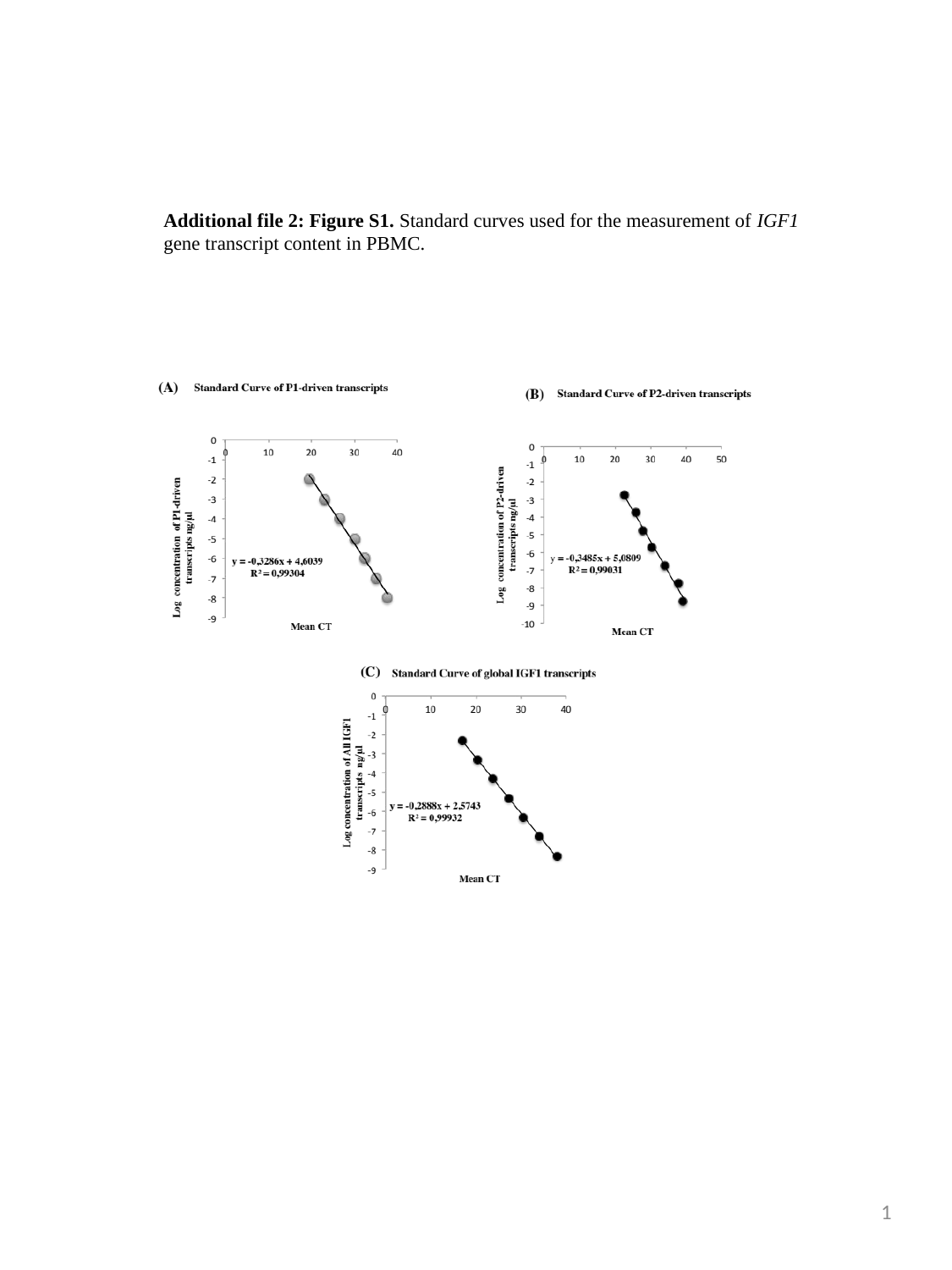

Additional file 2: Figure S1. Standard curves used for the measurement of IGF1 gene transcript content in PBMC.
1
